# Plasma Circular-RNA 0005567 as a Potential Marker of Disease Activity in Rheumatoid Arthritis

**DOI:** 10.3390/ijms25010417

**Published:** 2023-12-28

**Authors:** Marek Cieśla, Dorota A. Darmochwal-Kolarz, Konrad Kwaśniak, Anna Pałka, Bogdan Kolarz

**Affiliations:** 1 Institute of Medical Sciences, College of Medical Sciences, University of Rzeszow, 35-959 Rzeszow, Poland; ddarmochwal@ur.edu.pl (D.A.D.-K.); bkolarz@ur.edu.pl (B.K.); 2 Centre for Innovative Research in Medical and Natural Sciences, Medical College of Rzeszów University, 35-310 Rzeszow, Poland

**Keywords:** circular RNA, circ_0005567, rheumatoid arthritis

## Abstract

Circular RNAs (circRNAs) are noncoding molecules and are generated through back splicing, during which the 5′ and 3′ ends are covalently joined. Consequently, the lack of free ends makes them stable and resistant to exonucleases, and they become more suitable biomarkers than other noncoding RNAs. The aim of the study was to find an association between selected circRNAs and disease activity in patients with RA. A total of 71 subjects, 45 patients with RA and 26 healthy controls (HCs), were enrolled. In the RA group, 24 patients had high disease activity (DAS-28-ESR > 5.1) and 21 individuals were in remission (DAS-28-ESR ≤ 2.6). The cell line SW982 was used to evaluate the biological function of circ_0005567. The concentration of circ_0005567 in RA patients was elevated compared to HCs (median, 177.5 [lower–upper quartile, 83.13–234.6] vs. 97.83 [42.03–145.4], *p* = 0.017). Patients with high disease activity had a higher concentration of circ_0005567 than the control group (185.4 [112.72–249.25] vs. 97.83 [42.03–145.4], *p* = 0.015). In the cell line model, we found an association between circ_0005567 and miR-194-5p concentration and increased expression of mRNAs that may be related to cell proliferation. The plasma concentration of circ_0005567 may be a new potential biomarker associated with disease activity in patients with RA.

## 1. Introduction

Rheumatoid arthritis (RA) is one of the most prevalent chronic auto-inflammatory diseases leading to joint destruction, synovitis, and the manifestation of systemic immunity and inflammation [1]. It has been well documented that the pathogenesis of RA is multifactorial and that genetic, epigenetic, and environmental factors play a significant role in its development. However, the precise aetiology of RA remains unclear. Common laboratory markers used for the diagnosis of RA include the erythrocyte sedimentation rate (ESR), C-reactive protein (CRP), rheumatoid factor (RF), and anti-citrullinated protein antibodies (ACPA). To meet the classification criteria provided by The American College of Rheumatology (ACR) and the European League Against Rheumatism (EULAR) in 2010, it is necessary to perform laboratory tests [2], but most of them have limited specificity [3]. On the other hand, early diagnosis and effective treatment are important to prevent serious manifestations, especially that the disease has a progressive and irreversible course, often leading to disability and premature mortality [4]. Therefore, searching for new markers of disease development and severity may support a more effective diagnosis.

Recent genome-wide association studies have identified a single nucleotide polymorphism related to the development of RA, but these genetic variants may explain approximately 20% of susceptibility to disease [5]. From this point of view, epigenetic modifications, such as DNA or RNA methylation, histone protein alterations, or modifications in gene expression caused by noncoding RNAs (ncRNAs), emerge as crucial factors associated with RA pathogenesis, especially because nonprotein-coding genes occupy approximately two-thirds of the human genome [3]. Historically, ncRNAs were considered as genomic noise, but now there are undeniably molecules involved in the regulation of gene and protein expression. Moreover, it is already widely known that noncoding RNAs are important molecules involved in inflammatory responses and contribute to the pathogenesis of immune-related diseases, including RA [6].

Approximately 98% of RNAs are noncoding RNAs, including microRNAs, long noncoding RNAs, circular RNAs (circRNAs), and other types. Circular RNAs (circRNAs) are a subclass of ncRNAs that are formed during the pre-mRNA back-splicing process. CircRNAs may regulate gene expression levels in various ways, for example, by acting as a microRNA sponge or by interacting with RNA binding proteins. CircRNAs are divided mainly into three populations, exonic circRNAs, circular intronic RNAs, and exon-intron circRNAs [7]. The 3′ and 5′ ends of the transcript are covalently linked and form a loop structure, making it resistant to RNA exonucleases. Thus, circRNAs are biologically stable and may be considered as potential molecular markers of various diseases [8]. Importantly, circRNAs have been found in whole blood, peripheral blood mononuclear cells (PBMCs), serum/plasma, exosomes, and other body fluids, which makes the collection of material for laboratory tests easy and non-invasive. CircRNAs have previously been reported to be useful markers for the diagnosis of RA. Recent studies have shown that the quantity of circRNAs may be correlated with common laboratory markers of inflammation, such as ESR and CRP. Additionally, an association was found between their concentration and clinical characteristics, including, among others, disease duration or 28 joint disease activity scores (DAS28). Several studies evaluated the diagnostic impact of circRNAs. The combination of multiple circRNAs has been shown to be useful in distinguishing between RA patients from healthy controls [8,9].

The objective of the present study was to identify circRNAs whose plasma quantity is associated with the severity of well-established RA. Based on the current literature, we selected circRNAs that were related to various autoimmune disorders; however, there was not enough information about their association with RA severity.

## 2. Results

### 2.1. Patients

**Figure 1:**
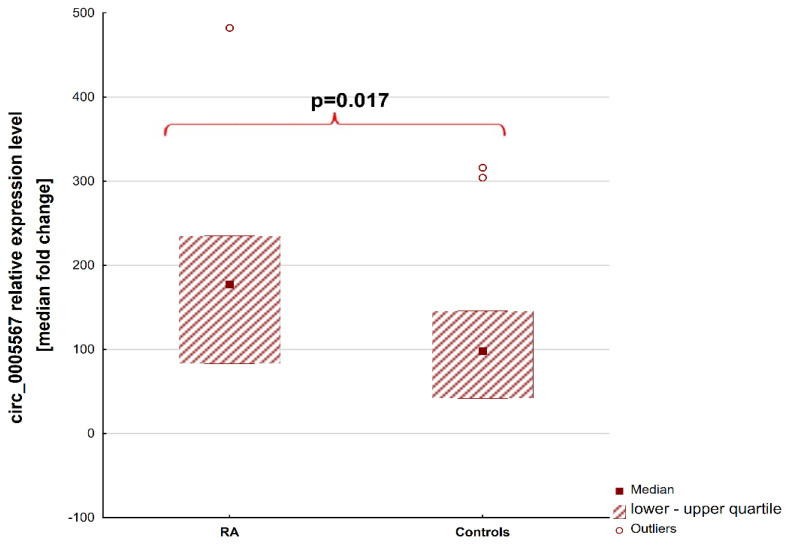
Differences in circ_0005567 levels in patients with rheumatoid arthritis and heathy controls. Abbreviations: RA—patients with rheumatoid arthritis.

The quantity of circRNAs was evaluated in patients with positive target genes amplification. The plasma concentration of circ_0005567 was detected in 39 samples from the RA (86.7%) group and 26 samples from the HC group (100%). Overall, the RA patients had an 81.4% higher plasma level of circ_0005567 than the control group (median, 177.5 [lower–upper quartile, 83.13–234.6] vs. 97.83 [42.03–145.4], *p* = 0.017). Figure 1 shows the differences in circ_0005567 levels in regard to patients with RA and healthy controls.

**Figure 2:**
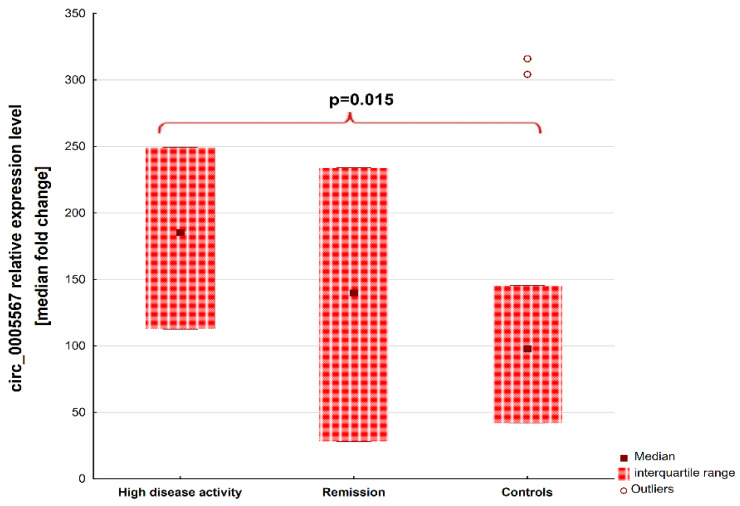
Differences in circ_0005567 levels in RA patients with high disease activity, remission, and healthy controls.

Differences in the plasma quantity of circ_0005567 were found between patients with RA with high disease activity (n = 20) and HCs (185.4 [112.72–249.25] vs. 97.83 [42.03–145.4], *p* = 0.015). These results are presented in Figure 2. No differences were observed between RA patients in remission (n = 19) and those with high disease activity (139.9 [28.01–234] vs. 185.4 [112.72–249.25], *p* = 0.68) as well as between patients in remission and controls (139.9 [28.01–234] vs. 97.83 [42.03–145.4], *p* = 0.44). The plasma concentration of Circ_0005567 was correlated with the following clinical variables: DAS28-ESR (rs = 0.34) and the number of swollen joints (rs = 0.32). However, it was not correlated with the number of painful joints (rs = 0.23); common laboratory markers of inflammation, ESR (rs = 0.18) and CRP (rs = −0.17); as well as autoantibody levels, RF (rs = 0.19) and ACPA (rs = 0.22).

**Figure 3:**
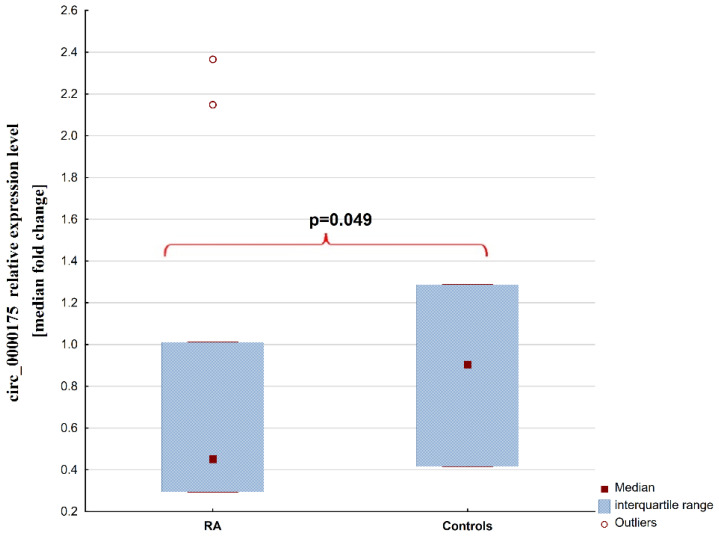
Differences in circ_0000175 levels in patients with rheumatoid arthritis and heathy controls. Abbreviations: RA—patients with rheumatoid arthritis.

Circ_0000175 was detected in 37 (82.2%) samples from the RA group and in 21 (80.8%) samples from HCs. Patients with RA have a decreased level of plasma circ_0000175 compared to controls (0.45 [0.29–1.01] vs. 0.9 [0.42–1.29], respectively, *p* = 0.049). These results are presented in Figure 3. No differences were observed when patients were divided into disease activity groups. Plasma circ_0000175 quantity was not correlated with any clinical variables: DAS28-ESR (rs = 0.13), number of swollen joints (rs = 0.17), number of painful joints (rs = 0.22), ESR (rs = 0.01), CRP (rs = −0.01), ACPA (rs = −0.14), and RF (rs = 0.01).

Both selected circRNAs were also evaluated in patients stratified by rheumatoid factor (RF) positivity. The RF-positive group (n = 23) and RF-negative group (n = 16) showed no significant differences in plasma concentration for both circRNAs (*p* = 0.49). Furthermore, the quantity of both studied molecules was not associated with ACPA positivity (ACPA-positive, n = 34 vs. ACPA-negative, n = 5; *p* = 0.93).

### 2.2. Circ_0005567 Silencing

**Figure 4:**
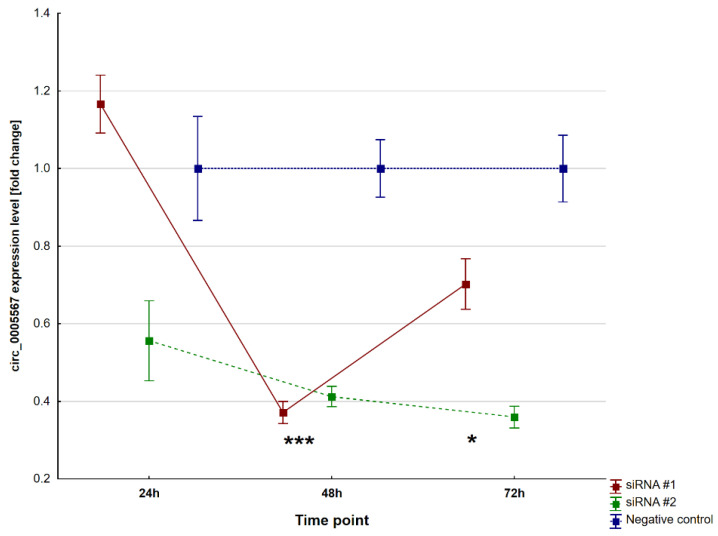
The relative expression level of circ_0005567 after siRNAs transfection. Abbreviations: * both *p*-values < 0.05; *** both *p*-values < 0.0001.

Circ_0005567 expression levels were normalised with reference to the negative control, and the expression in the negative control was also divided by its value to obtain the unit expression value. Both the siRNA#1 and siRNA#2 molecules effectively silenced the expression levels of circ_0005567 after 48 h (*p* < 0.0001 and *p* ≤ 0.0001, respectively) and 72 h (*p* = 0.01 and *p* < 0.0001, respectively). The effectiveness of circ_0005567 silencing is presented in Figure 4.

**Figure 5:**
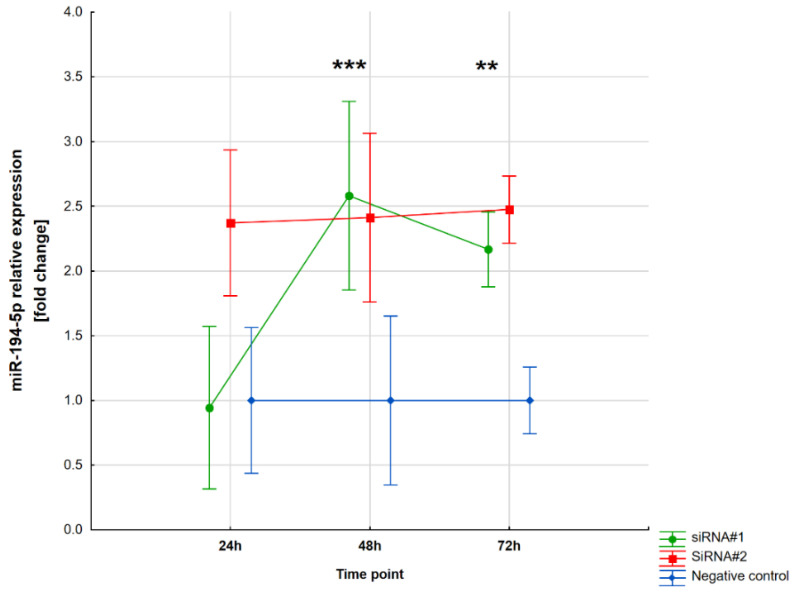
The relative expression level of miR-194-5p after siRNAs transfection. Abbreviations: *** both *p*-values < 0.001 and ** both *p*-values < 0.01.

The relative expression of miR-194-5p was also determined in the same cells. Both siRNA#1 and siRNA#2 were responsible for the upregulation of miR-194-5p at both time points, after 48 h (*p* < 0.001 and *p* = 0.001, respectively) and 72 h (*p* = 0.002 and *p* = 0.0001, respectively). The miR-194-5p dysregulation after transfection is presented in Figure 5.

**Figure 6:**
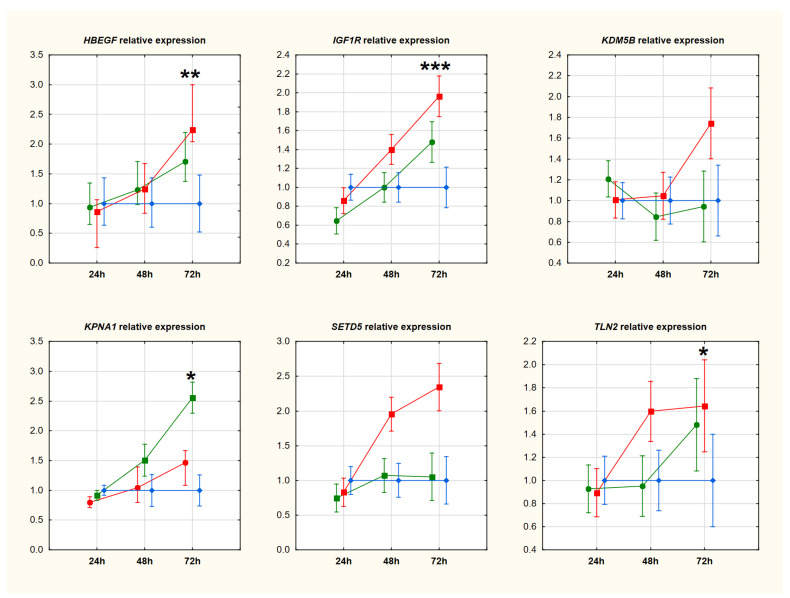
Relative expression of genes selected from in silico analysis. Data were evaluated by ANOVA with repeat measurements and presented by mean expression levels with 95% confidence intervals. The red line is associated with cells transfected with siRNA#1, the green line with siRNA#2, and the blue line is related to the negative control. Abbreviations: * *p*-value < 0.05; ** *p*-value < 0.01; *** *p*-value < 0.001.

**Figure 7:**
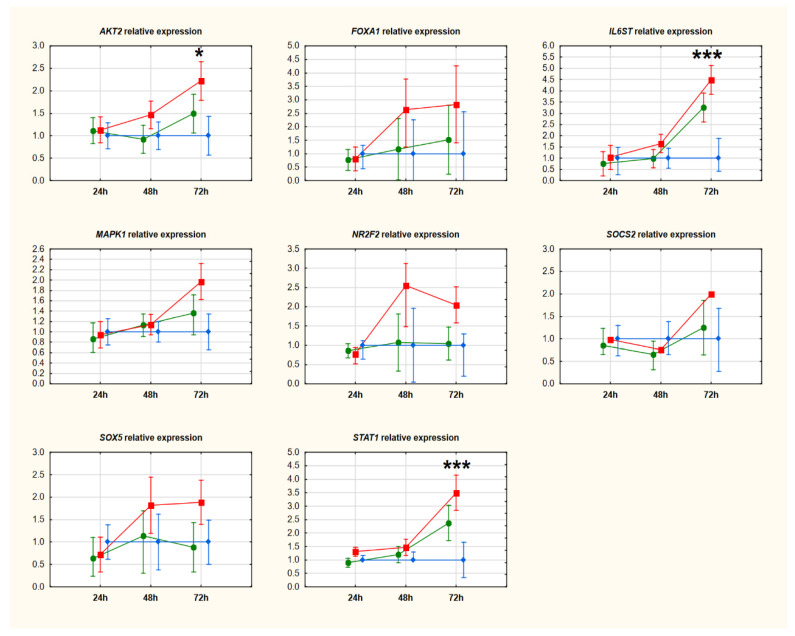
Relative expression of genes selected from analysis of the literature. Data were evaluated by ANOVA with repeat measurements and presented by mean expression levels with 95% confidence intervals. The red line is associated with cells transfected with siRNA#1, the green line with siRNA#2, and the blue line is related to negative control. Abbreviations: * *p*-value < 0.05; *** *p*-value < 0.001.

After downregulating circ_0005567, we observed an upregulation of miR-195-5p, which suggests that circ_0005567 may act as a sponge and miR-194-5p may be its potential molecular target. We assessed the expression of mRNAs of genes potentially regulated by this miR, six from in silico analysis and eight from the literature search. None of the transcripts tested showed a significant reduction in expression levels compared to the negative control. Seven of them (from in silico analysis: *HBEGF*, *IGF1R*, *KPNA1*, and *TLN2;* and from the literature search: *AKT2*, *IL6ST*, and *STAT1)* showed significant upregulation 72 h after transfection. Detailed data are presented in Figure 6 (genes selected from in silico analysis) and Figure 7 (genes selected from the literature search).

## 3. Discussion

The current study is the first to demonstrate a novel finding that circ_0005567 may be associated with disease activity in patients with RA. The study further indicates that decreased expression of circ_0005567 and increased expression of miR-194-5p may be associated with the pathogenesis of RA based on the SW982 cell line model. It is possible that the investigated circular RNA acts as a sponge, capturing the mentioned miR leading to a reduction in its concentration.

Circular RNAs (circRNAs), serving as stable forms of noncoding RNAs (ncRNAs) implicated in the regulation of gene expression, are abundantly present in various tissues, including blood. This makes them a crucial focus of contemporary research into the pathogenesis of various diseases. Consequently, circRNAs emerge as promising candidates for modern markers of rheumatoid arthritis (RA) [10]. Of the circRNAs tested (circ_0000175; circ_0044235; circ_0005198; circ_0005008; circ_0003146; circ_0005567; circ_0003304; circ_0060918; circ_0000039; circ_0087932; circ_0083738; circ_0000906; circ_0002453—identified for our study based on the literature review), only circ_0000175 and circ_0005567 exhibited differences in their plasma expression levels between the rheumatoid arthritis (RA) and healthy control (HC) groups.

We confirmed the importance of circ_0000175 in RA within the evaluated group of patients with established RA, which lasted 10 years on average. Yujie Gao et al. indicated the importance of its examination in blood in new-onset RA [1]. They also previously studied its expression in PBMCs and confirmed that this circRNA plays a significant role in RA. In this compartment, the level of circ_0000175 was found to be decreased [11].

Our results indicate that this circRNA plays a consistent role in the pathogenesis of RA throughout the course of the disease over consecutive years and its level is higher than in the control group. Investigations into circ_0000175 suggest its involvement in the pathogenesis of other autoimmune diseases, including systemic lupus erythematosus (SLE) and its correlation with platelet count (PLT) or peripheral neuropathy [12]. Furthermore, there are reports indicating an association of this circRNA with rheumatoid arthritis (RA) activity, assessed by the platelet-to-lymphocyte ratio (PLR). However, we were unable to demonstrate an association of circ_0000175 with RA activity in the form of DAS28, tender joint count, ESR, and swollen joint count. Exploration of circ_0000175 in rheumatoid arthritis (RA) across different compartments indicates that its levels may vary depending on the site of measurement. The data indicate an elevation in plasma and a decrease in peripheral blood mononuclear cells (PBMCs) in RA. A similar phenomenon was observed for miR-155, as documented in one of our earlier publications [13].

Regarding circ_0005567, whose plasma levels were statistically significantly higher in the rheumatoid arthritis (RA) group compared to the healthy control (HC) group in our study, there are limited reports on its role in the disease. The existing literature presents certain contradictions, contributing more to uncertainty than offering clear evidence.

On the basis of in silico studies, a potential target of this circRNA, namely miR-492, was postulated. This hypothesis was substantiated in a publication by Zhang J et al. [14], wherein the authors suggest that the observed overexpression of circ_0005567 (also noted in our study) may promote M2-type macrophage polarisation through the miR-492/SOCS2 axis. It should be noted that this study involved tissues obtained from patients with osteoarthritis (OA). In another publication by the same research centre, the authors highlight the association of circ_0005567 with miR-495 and its ability to inhibit IL-1β-induced chondrocyte apoptosis and activate the autophagy process in a chondrocyte cell culture [15]. However, no such data are available in the literature concerning RA. It should also be mentioned that circ_0005567 is encoded by the EGF receptor pathway substrate 15 gene (*EPS15*). This gene was previously studied with respect to gene expression profiling in RA. Differential expression of the *EPS15* gene was reported and it was indicated that the gene is located in a previously identified region associated with susceptibility to RA [16]. Another study identified a potential role for the *EPS15* gene in white blood cells in predicting response to infliximab in rheumatoid arthritis [17]. The circ_0005567 molecule seems to be an important marker not only related to the disease’s development and severity, but it may also be a pharmacogenetic marker, although this requires further research.

In our study, we used cell cultures to validate the relationship between circ_0005567 and miR-194-5p levels. The human synovial sarcoma cell line (SW982) is the most commonly used model to study the pathogenesis of RA, with particular emphasis on synovitis. SW982 has been selected as a cell model due to its high proliferation rate, compared to human fibroblasts [18,19]. In the study by Khansai et al. [18], TNF-α was used as an acute inflammatory stimulant in human fibroblasts and the SW982 line to induce inflammation development in RA. It was observed that both cell types showed a quite similar response. Under acute inflammatory conditions, both human fibroblast cells and the SW982 cell line responded by increasing the expression of IL-6, IL-8, IL-1β, and TNF-α mRNA expression. Due to the fact that our study showed that the concentration of circ_0005567 is mainly increased in patients with high disease activity (DAS28-ESR index > 5.1), it seems justified to use SW982 as a model that can help understand the pathogenesis of RA.

Recent studies suggest that miR-194 plays a critical role in various biological pathways, including tumorigenesis, the pathogenesis of proliferative vitreoretinopathy [20], stimulating osteogenesis, and inhibiting adipogenesis [21] and depression [22]. However, the most important thing seems to be that the miR-194-5p molecule may have a pro-inflammatory effect. The study conducted by Wang et al. [22] showed that the decreased expression levels of miR-194-5p in LPS-induced astrocytes may be associated with decreased proliferation and migration. Furthermore, miR-194 was responsible for the inhibition of COX2 and pro-inflammatory cytokines, such as transforming growth factor β, TNF-α, IL-1β, and IL-6 [23]. Another study confirmed that miR-194 may decrease inflammation by suppressing the TGF-β/SMAD pathway [24]. Upregulated serum concentrations of hsa-miR-194-5p and miR-432-5p were found to be associated with relapse in patients with RA, with a notable link to the clinical activity of RA, particularly in the case of miR-194-5p overexpression [25]. In this study, we did not confirm that elevated levels of miR-194-5p may play an anti-inflammatory role.

In this study, it was observed three genes related to RA pathogenesis were the most affected by overexpression, including *IL6ST*, *STAT1,* and *IGF1R*. It seems that increased expression of these genes may be related to the specificity of the cell line used. On the other hand, a significant increase in expression occurred 72 h after transfection and was not observed in the negative control. Upregulation of IGF1R in leukocytes was associated with general inflammation, as it was related with increased expression of NF-kB and serum levels of IL6 and ESR [26]. Furthermore, IL6 and gamma-interferon IFNγ are responsible for activation via the Jak/STAT signalling pathway and IL6 is involved in the activation of both the STAT1 and STAT3 molecules [27]. Other studies confirmed that STAT1 and other immune-related genes were upregulated in patients with RA [28]. *IL6ST* encoded the protein gp130 which is a signal transducer related to many cytokines, including IL6. The binding of IL6 to its receptor causes the dimerisation of two gp130 proteins, thus transmitting the signal into the cells. In our study, we expected that an increase in miR-194-5p concentration would be associated with a decrease in the expression of the targeted genes selected for analysis. However, this did not occur and we can only speculate on what it is related to. It can be concluded that the overexpression of miR-194-5p likely leads to the activation of compensatory mechanisms [29], which may promote the expressions of genes related to proliferation. Both the *STAT1* and *IGF1R* genes are also responsible for cell proliferation [30]. In addition, miR-194-5p has been described as a molecule that influences cell proliferation and migration processes. The study by Yang et al. [31] showed that the downregulation of miR-194-5p inhibits cell proliferation, migration, and invasion in breast cancer cells. Chi et al. presented similar findings. They showed that increased expression of miR-194-5p through the circPVRL3/miR-194-5p/SOCS2 axis induces proliferation [32]. Other conclusions were presented by Zhao et al. [33]. In their study, the increased expression of miR-194-5p was related to the downregulation of STAT1 and significantly inhibited proliferation and invasion and promoted the apoptosis of mice ectopic endometrial cells. In our study, SW982 has been selected as a cell model due to its high proliferation rate, in comparison to human fibroblasts, thus this may be a solution to why we observed the increased expression of genes related to proliferation. From our perspective, increased expression of genes such as *STAT1* and *IGF1R* compared to the negative control may indicate that miR-194-5p may be more involved in stimulating proliferation than inhibiting inflammation. This conclusion requires further verification, because in this study, only the primary effect between circ_0005567 and miR-194 was examined and mRNA expression was evaluated as a derivative of this process. It should be noted that both circ_0005567 and miR-194-5p interact with many genes, so determining the relationship between circRNA, miR, and mRNA is not simple. The study conducted by Fernandez-Ruiz et al. [25] showed that approximately 2600 genes were downregulated and approximately 2400 genes were upregulated after transfection of the 22Rv1 cell line with miR-194. This shows that this molecule can largely influence a large part of the transcriptome, which is why there are many publications on the impact of this molecule on individual genes.

The most important observation of this study is the relationship between circ_0005567 and miR-194-5p, which may suggest an important role of circ_0005567 in the pathogenesis of RA. To confirm our results, further studies should be conducted in human fibroblast cells to better understand the role of circ_0005567 and miR-194-5p in the pathogenesis of RA and their prognostic value.

We showed the association between disease activity and plasma concentration of circ_0005567. We focused on plasma determinations because we are looking for markers of disease activity that can be determined in biological material, the collection of which is not invasive for the patient. Moreover, plasma is a biological material that is easy to obtain, easy to store for a long time, and free of ribonucleases that can cause degradation of the genetic material. The search for new markers of disease activity, including inflammation, is important from the point of view of the treatment of patients with RA. Previous studies have shown that approximately half of RA patients with normal CRP levels have histological signs of synovitis, and almost three-quarters of patients in remission show inflammation within the tissues based on DAS28-CRP [34]. Finally, DAS28-CRP is able to detect histological inflammation with approximately 92% sensitivity and only 6% specificity [34]. Another study showed that approximately one third of RA patients had normal levels of CRP, ESR, and RF [35]. Moreover, routinely used serological markers including RF and ACPA also have their limitations. RF sensitivity in the advanced phase of the disease can be as high as 79% and overall specificity does not exceed 84%, and ACPA, measured using an anti-cyclic citrullinated peptide antibody test, has a sensitivity and specificity of 70% and 96%, accordingly [36]. For these reasons, there is a need to obtain new supporting molecular markers of disease development and activity.

In this study, we found only an association between the plasma concentrations of circ_0005567 and DAS28, but there was no correlation with the level of commonly used laboratory markers of inflammation, such as ESR and CRP. From our point of view, it is prospectively important because it may indicate that circ_005567 is an independent but supportive marker associated with disease severity. The diagnostic potential of circRNAs in RA was previously evaluated [9]. The study showed that simple circRNA can distinguish between RA and healthy people. The sensitivity of the circRNAs was in the range of 50.9% to 83.3% and the specificity was 52% to 88.6%. The implementation of a comprehensive analysis involving multiple molecules has the potential to enhance the sensitivity and specificity of the test, enabling a clear distinction between RA patients and healthy individuals. For example, the use of a combination of two molecules, circ_0000175+circ_0008410, increases the sensitivity and specificity to 93.1% and 93.33%, respectively. In this study, we demonstrated for the first time that circ_0005567 may be a potential marker of disease activity in well-established RA (median disease duration was about 10 years).

Our study did not demonstrate the circRNA–miR–mRNA relationship (downregulation of mRNA). We knocked down the expression of circ_0005567 alone and did not additionally knockdown the miR-194-5p molecule. There are many publications in the literature that describe the relationship between miR-194-5p and target mRNAs. We wanted to check whether dysregulation of the circ_0005567 molecule could directly cause gene dysregulation at the mRNA level. It seems that this effect could not be achieved because compensation mechanisms in the cells were activated. However, this was not the main goal of our study. We focused on the identification of a circular RNA molecule that is associated with disease activity and can be measured in plasma. Further research is needed to determine other relationships.

Our study has certain limitations. Firstly, we used immortalised cells from the SW982 cell line. To better understand the biological role of circ_0005567 in RA pathogenesis, human fibroblasts should be used. The second limitation is the relatively small sample size; thus, further analysis on a larger cohort is necessary to confirm obtained results, especially the conclusions regarding usefulness of circ_0005567 as an additional marker distinguishing patients with RA and healthy controls as well as distinguishing RA from other autoimmune rheumatic diseases, for example, SLE or OA. Moreover, subjects were enrolled from one medical centre, thus an independent validation cohort is needed. We compered well-established RA (median disease duration was about 10 years) to healthy controls, but further studies including early RA and pre-RA patients are necessary to better understand the role of circ_005567 in disease development and pathogenesis. It should be noted that we tested statistical relationships for patients with positive amplification of these molecules. This is due to the fact that few patients did not express the tested markers, which, in our opinion, interferes with drawing correct conclusions regarding this molecule. This was also due to the fact that in the cell line model, we observed a reduction in circ_0005567 expression but not its complete knockout. Our conclusions should be considered valid only for patients with RA with positive amplification of the tested targets. Additional studies are necessary to determine whether the lack of expression of this marker is clinically significant or whether it may be an artifact of the PCR reaction caused, for example, by an insufficient amount of genetic material in the sample, the presence of reaction inhibitors, or the treatment used.

Numerous ongoing studies aim to identify circRNAs that can serve as disease markers, predictors of treatment response (both favourable and unfavourable), and more. Our publication presents some preliminary findings on the circRNAs under investigation, contributing to the ongoing discussion. Nevertheless, further research is essential to refine and validate our results. RNAs, especially noncoding RNAs (for example micro-RNAs), show a low specificity to one disease, thus the use of a single molecule to diagnose the disease development or to evaluate its activity may be difficult. Rather, circRNAs should be considered as an additional supporting method, independent of the common laboratory serological markers and parameters related to inflammation. Due to their back splicing, circular RNA molecules are resistant to the action of nucleases, which makes them more resistant to degradation, unlike linear forms (for example mRNA, long noncoding RNAs). This fact makes them stable molecules and suitable candidates for new molecular markers. In conclusion, the plasma concentration of circ_0005567 may be considered as a new supporting molecular marker related to disease activity.

## 4. Materials and Methods

### 4.1. Patients

A total of 71 subjects (45 patients with RA, aged 50.29 ± 13.42 and 91.1% women; and 26 healthy controls (HCs), aged 51.81 ± 7.79 and 80.8% women) were enrolled to the study. RA patients were selected based on current disease activity. In the RA group, 24 patients with high disease activity (DAS-28-ESR > 5.1; 53.3%) and 21 individuals in remission (DAS-28-ESR ≤ 2.6; 46.7%) were included in the circRNA analysis. The study was conducted in accordance with the Declaration of Helsinki, the protocol was approved by the Bioethics Board at the Medical University in Lublin, protocol number KE-0254/7/2016, and patients provided written informed consent. RA diagnosis was made according to the 2010 ACR/EULAR or 1987 ACR criteria for classification depending on the time of diagnosis. Infection or severe illness during hospitalisation were the exclusion criteria. Detailed characteristics of the patients and controls are presented in Table 1 and Table 2.

### 4.2. Circular-RNA Analysis in Plasma Samples

Circular RNAs (circRNAs) were found based on the current literature and targets with established concentrations in plasma were selected. These were the following molecules: circ_0000175; circ_0044235; circ_0005198; circ_0005008; circ_0003146; circ_0005567; circ_0003304; circ_0060918; circ_0000039; circ_0087932; circ_0083738; circ_0000906; and circ_0002453 (accession numbers according to Circular RNA Interactome available from https://circinteractome.nia.nih.gov; accessed on 6 February 2022) [37,38].

Blood samples with EDTA were collected and centrifuged at 2500 rpm for 10 min at room temperature to obtain plasma. After that, the samples were stored at −80 °C until analysis. Total RNA extraction has previously been described [39]. Briefly, plasma RNAs were extracted using the miRCURY RNA Isolation Kit—Biofluids (Exiqon, Vedbaek, Denmark) according to the manufacturer’s instructions, but 300 µL of plasma was used for the extraction of RNAs.

To perform reverse transcription of circRNAs in complementary DNA (cDNA), the PrimeScript RT Reagent Kit (Takara Bio, Kusatsu, Japan) was used according to the manufacturer’s recommendation. Both the oligo dT and random hexamer primers were used to perform the reaction and 4.5 µL of total RNA was reverse transcribed in a total volume of 15 µL. After that, cDNA was stored at −20 °C until PCR. Before PCR, cDNA was 2-fold diluted using the easy dilution buffer supplied with the kit.

First, the screening was performed. Three samples from the RA group and the HC group were randomly selected and the plasma concentration of circRNAs was evaluated. The reaction was carried out using the 2× SG master mix (Eurx, Gdańsk, Poland) in the COBAS z480 real-time PCR system under the thermal cycling conditions given in the mix manual, in 45 amplification cycles and with an annealing step at 59 °C for 30 s and elongation at 72 °C for 30 s. PCR was followed by a melt curve analysis. Divergent primers were designed using an online tool provided by the Circular RNA Interactome database, available as previously specified. Targets that showed expression in at least two samples in each group and Ct values below 40 were also included in the study. The following circRNAs met these criteria: circ_0000175 and circ_0005567. PCR products’ specificity was also evaluated by 2% agarose gel electrophoresis. Plasma concentration for both targets was evaluated in all individuals. After optimisation of the qPCR reaction, the plasma concentration of circ_0000175 was assessed using TB Green Premix Ex Taq II (Takara Bio, Kusatsu, Japan) and the quantification of circ_0005567 was evaluated using 2× SG mix (Eurx, Gdańsk, Poland). Both reactions were performed under the following conditions: 45 amplification cycles, annealing step at 55 °C for 30 s, and elongation at 72 °C for 1 min. The glycoseraldehyde 3-phosphate dehydrogenase (*GAPDH*) gene was used for data normalisation and primer sequences were previously described [40]. Primers’ final concentrations were as follows: 200 nM for both *GAPDH* and circ_0005567 and 450 nM for circ_0000175. PCR was followed by a melt curve analysis. All samples were evaluated in triplicate. CircRNAs’ concentration was considered positive when there was at least one amplification signal in two replicates and the Ct value was below 40. Detailed characterisation of primers and targeted circRNAs is presented in Supplementary Table S1. Data were analysed using the Advanced Relative Quantification method (LightCycler 480 SW, version 1.5.1.62 SP2—UDF v.2.0.0, Roche, Ludwigsburg, Germany), with the maximum second derivative selected as the calculation model. The results are presented as a normalised ratio.

### 4.3. Cell Culture and Transfection

The SW982 (HTB-93^TM^) cell line was purchased from the American Type Culture Collection (ATCC; Manassas, VA, USA) and cultured in Dulbecco’s Modified Eagle’s Medium (DMEM; Thermo Fisher Scientific, Waltham, MA, USA) supplemented with 10% foetal bovine serum (FBS: EurX, Gdańsk, Poland) and 1% penicillin/streptomycin (Biowest, Nuaillé, France). The cell line was grown at 37 °C, in a humidified atmosphere, with 5% CO_2_. The culture medium was refreshed every 2–3 days. Once the cells reached 70 to 80% confluence, they were removed using the cell scraper. The cells were seeded in flat-bottom 24-well culture plates and allowed to attach and grow for 48 h before treatment. Transfection was performed according to the manufacturer’s instructions using small-interfered RNAs (siRNAs) and RNAiMAX reagent (Thermo Fisher Scientific, Waltham, MA, USA). The assay contained a positive control against the *GAPDH* gene and a negative control (Thermo Fisher Scientific, Waltham, MA, USA, cat. no. 4390849 and AM4611, respectively). The experiment was carried out in three biological replicates. SiRNAs were designed using the online tool provided by the Circular RNA Interactome database, available as previously provided, and two siRNAs targeting one transcript were designed. The detailed characterisation of siRNAs is provided in Supplementary Table S2. Due to the lack of statistical relationships between the disease activity and the level of circ_0000175, it was decided to carry out the procedure only for circ_0005567.

### 4.4. Interactions between circRNA, microRNA, and mRNA

After 24 h, 48 h, and 72 h, the cells were isolated for RNA extraction. RNA was extracted using a Universal RNA/miRNA Purification Kit (EurX, Gdańsk, Poland) according to the manufacturer’s protocol. In the first stage, the effectiveness of expression silencing for circ_0005567 was tested for two designed siRNA molecules. For this purpose, 100 ng of total RNA was reverse-transcribed using the SuperScript Vilo cDNA Synthesis kit (Thermo Fisher Scientific, Waltham, MA, USA) according to the manufacturer’s recommendation. After that, cDNA was stored at −20 °C until downstream analysis. Before the polymerase chain reaction (PCR), cDNA was diluted 5 times and reactions were performed using SG onTaq qPCR Master Mix (Eurx, Poland) in the QuantStudio 5 Real Time PCR System (Thermo Fisher Scientific, Waltham, MA, USA) under the thermal cycling conditions given in the mix manual, in 40 amplification cycles and with an annealing/elongation step at 60 °C for 1 min. Melt curve analysis was performed after each reaction plate. The *GAPDH* gene was used as a reference gene, as previously described [40]. After confirming that the studied circ_0005567 molecule is silenced by both designed siRNAs with reference to the negative control, the relationship between the targeted circRNA and possible microRNAs was evaluated. Using the prediction tool (available at https://circinteractome.nia.nih.gov; accessed on 30 March 2022), the following set of microRNAs (miRs) that potentially interact with the studied molecule was identified: hsa-miR-194; hsa-miR-620, hsa-miR-1270, hsa-miR-383, hsa-miR-579, and hsa-miR-548c. The detailed characterisation of primers and target miRs is presented in Supplementary Table S3. To perform the reverse transcription of miRNAs to complementary DNA (cDNA), the miRCURY LNA RT Kit (Qiagen, Hilden, Germany) was used. An amount of 10 ng of total RNA was transcribed according to the manufacturer’s recommendation. After that, cDNA was stored at −20 °C until downstream analysis. Before PCR, cDNA was 10-fold diluted and the reaction was carried out using TB Green Premix Ex Taq II (Takara Bio, Kusatsu, Japan) in the QuantStudio 5 Real Time PCR System (Thermo Fisher Scientific, Waltham, MA, USA) under the thermal cycling conditions given in the mix manual, in 45 amplification cycles, with an annealing step at 58 °C for 30 s, and an elongation step at 72 °C for 30 s. Melt curve analysis was performed after each reaction plate. Primers complementary to selected miRs were designed by miRprimer2 software [41]. U6 small nuclear RNA was used for data normalisation and sequences of primers were designed using Primer3 software (available from https://bioinfo.ut.ee/primer3-0.4.0/; accessed on 31 March 2022) [42,43]. The specificity of the U6 primers was tested in silico using the Primer-BLAST online tool, available from https://www.ncbi.nlm.nih.gov; accessed on 31 March 2022 [44], and the PCR product was visualised in 2% agarose gel.

As a result of the miRs expression analysis, only miR-194-5p was found to be the most suitable molecule for further evaluation. The interactions between miRs and messenger RNAs (mRNAs) were tested in silico using the online tool miRWalk, available from http://mirwalk.umm.uni-heidelberg.de; accessed on 3 October 2022 [45]. The following filtering parameters were applied: minimum binding probability 0.9; binding site: 3′UTR, CDS, and 5′UTR as well as validated interactions—the results with the interactions of Targetscan [46,47] and the Mirdb database [48] and Mirtarbase [49] were considered for further analysis. The results showed that 9 possible transcripts could interact with miR-194-5p and these were the following mRNAs: *ITGA9*, *TJAP1*, *SETD5*, *ITSN1*, *KPNA1*, *HBEGF*, *KDM5B*, *TLN2*, and *IGF1R*. After the literature analysis, the *TJAP1* gene was excluded from further evaluation due to the lack of association with RA pathogenesis. The primers were designed using the Primer-BLAST online tool (available as mentioned above; accessed on 3 October 2022), except primers related to the *IGF1R* gene whose sequences were previously described [40]. Additionally, a literature search was performed to identify genes that were previously indicated to downregulate mRNA expression by miR-194-5p. The following genes were selected: *SOCS2*, *MAPK1*, *STAT1*, *FOXA1*, *AKT2*, *SOX5*, *IL6ST*, and *NR2F2* [32,33,50,51,52,53,54,55]. The detailed characterisation of primers is presented in Supplementary Tables S4 and S5.

Primers were designed using the Primer-BLAST online tool (available and accessed as was stated above) and their in silico specificity was evaluated. An amount of 100 ng of total RNA was reverse transcribed using the SuperScript Vilo cDNA Synthesis kit (Thermo Fisher Scientific, Waltham, MA, USA), as described above, and before PCR, cDNA was diluted 10 times. Reactions were performed as was stated in a description of the effectiveness of expression silencing for circ_0005567. The PCR products were visualised on a 2% agarose gel. At this step, both *ITGA9* and *ITSN1* were excluded due to unspecified amplification.

### 4.5. Statistical Analysis

The data distribution was evaluated using the Shapiro–Wilk W test. Quantitative values with a normal distribution were presented as mean ± SD, otherwise medians [lower–upper quartile] were given. Differences between two independent groups were compared using Student’s *t* test; otherwise, the Mann–Whitney U test was used. The Kruskal–Wallis ANOVA and post hoc multiple comparison analysis were used to assess differences between three groups (healthy controls vs. high disease activity vs. remission). The relationship between two continuous variables was analysed using Spearman’s correlation. For comparison of expression in 24 h/48 h/72 h of culture, repeated measures ANOVA with Fisher’s NIR post hoc test was used. A *p*-value less than 0.05 was considered statistically significant. The analysis was performed with STATISTICA Version 13 (Dell Inc. 2016).

## 5. Conclusions

The plasma concentration of circ_0005567 may be a new potential biomarker associated with disease activity in patients with RA.

## Figures and Tables

**Table 1:** Characteristics of subjects enrolled to the study.

Characteristics	RA Overall n = 45	Controls n = 26	*p*-Value RA Overall vs. HC
Age, years	50.3 ± 13.4	51.8 ± 7.8	0.6
Females	41 (91.1)	21 (80.8)	0.37
Disease duration, years	10.5 [5.5–16.5]	n/a	n/a
RF-positive	29 (64.4)	none	n/a
ACPA-positive	40 (88.9)	none	n/a
ESR, mm/h	15 [8–51]	16 [7.5–21.5]	0.32
DAS28	5.22 [2.11–6.12]	n/a	n/a
CRP, mg/dL	5.84 [0.44–17.47]	0.6 [0.2–1.97]	**0.009**
Number of swollen joints	3 [0–7]	n/a	n/a
Number of painful joints	4 [1–12]	n/a	n/a
VAS PGA	39 [9–70]	n/a	n/a
VAS PhGA	30 [5–60]	n/a	n/a

Data are presented as mean ± SD; number (%) or median [interquartile range]. Abbreviations: ACPAs, anti-citrullinated protein antibodies; CRP, C-reactive protein; DAS28, disease activity score; ESR, erythrocyte sedimentation rate; HCs, healthy controls; RA, rheumatoid arthritis patients; RF, rheumatoid factor; VAS PhGA, visual analogue scale physician global assessments; VAS PGA, visual analogue scale patient global assessments. Differences between two independent groups, according to the data distribution, were evaluated using Student’s *t* test or Mann–Whitney U test. Significant differences (*p*-value < 0.05) are bolded.

**Table 2:** Characteristics of patients with rheumatoid arthritis.

Characteristics	RA in High Disease Activity, n = 24	RA in Remission n = 21	*p*-Value
Age, years	53.5 ± 13.05	46.7 ± 13.2	0.09
Females	21 (87.5)	20 (95.2)	0.7
Disease duration, years	10.5 [8–17.5]	10 [3–16.5]	0.37
RF-positive	19 (79.2)	10 (47.6)	0.058
ACPA-positive	22 (91.7)	18 (85.7)	0.87
ESR, mm/h	49.5 [28–67]	7 [2–10]	**<0.0001**
DAS28	6.1 [5.4–6.5]	2.1 [1.8–2.4]	**<0.0001**
CRP, mg/dL	16.7 [8.5–24.4]	0.4 [0.2–1.6]	**<0.0001**
Number of swollen joints	6.5 [5–10]	0 [0–1]	**<0.0001**
Number of painful joints	11.5 [7–14]	1 [0–2]	**<0.0001**
VAS PGA	69 [51.5–77.5]	9 [3–15]	**<0.0001**
VAS PhGA	60 [49–70]	5 [2–15]	**<0.0001**

Data are presented as mean ± SD; number (%) or median [interquartile range]. Abbreviations: please refer to Table 1. Differences between two independent groups, according to the data distribution, were evaluated using Student’s *t* test or Mann–Whitney U test. Significant differences (*p*-value < 0.05) are bolded.

## Data Availability

Data generated and analysed during the current study are available in the manuscript and in the Supplementary Material.

## Supplementary Materials

The following supporting information can be downloaded at https://www.mdpi.com/article/10.3390/ijms25010417/s1.
