# Supplementary material for: Plasma Circular-RNA 0005567 as a Potential Marker of Disease Activity in Rheumatoid Arthritis
Source: Int J Mol Sci. 2023 Dec 28;25(1):417. doi: 10.3390/ijms25010417 (PMC10779327; doi:10.3390/ijms25010417)
Supplement: Supplementary file 1 [file ijms-25-00417-s001.zip › ijms-2757271-supplementary.pdf]

Supplementary materials.

Plasma circular-RNA 0005567 as a potential marker of disease activity in rheumatoid arthritis.

Cieśla M. et al.

Table S1. Characterization of primers used for circRNAs evaluation.

Circ_RNA ID*	Primer sense 5′→3′	Primer antisense 5′→3′	Gene
**hsa_circ_0000175**	**GCCCATTTTCCCCAGACCTAC**	**GGAACTGCCACAGGGTGATA**	**ELK4**
hsa_circ_0044235	TGCTTTACTCTTTCATTGTTGGGAC	GCTTCTTCTGAGTGTACTTCTGC	CDC27
hsa_circ_0005198	TGGTGGAGCTTCAGTGTTCG	TTCTTCTGCTGCTGAGGTAAGT	PARP4
hsa_circ_0005008	AGTGGTCGTGGTGGCTTT	ACCGAAGTTGTCATTCCCA	TCONS_l2_00009739
hsa_circ_0003146	CGCAAGCTCAACGACCTG	CGCATCAAAGAGCAGGATGA	EHD2
**hsa_circ_0005567**	**TGTAGACTCAAGTTCGCTGAC**	**GCCAATTCTTCTTCGTAAGTGGAG**	**EPS15**
hsa_circ_0003304	GCAGCAAAAGTTGGAGTGAA	CCGAAAGTCATAAACCAGGAA	AZIN1
hsa_circ_0060918	CTTGTGTGCCTGCTGGTAGT	AGCATTAGTTCTCTTTGTCTCCATT	ZNF217
hsa_circ_0000039	GCTACAAGCACACCACTTCC	ACCCAAAGAATAGGAGAAGCCA	YTHDF2
hsa_circ_0087932	CATTGTCGGGCTTGGAAA	CTGCTCTCAAGTAGCGTTCAGT	C9orf5
hsa_circ_0083738	GTTGTTGGAATGGATCTGACAGC	CTCCTGCTCCTGTTGAAAGATG	PPP2R2A
hsa_circ_0000906	GGTCAAAGGTTCTGGTGTCG	GATGTAGCTCCCGAAGCAATG	TMEM38A
hsa_circ_0002453	ACAGCTCATTAAAAGGCACCA	GTCACACAGCAAGTTGGACAC	RAD18

**According to nomenclature provided by: https://circinteractome.nia.nih.gov.*

Table S2. Characterization of siRNAs used for knockdown of circ_0005567.

	Name	Sequence	3' DNA overhang
**Set 1**	siRNA_0005567_sens1	5'-UCCUUUUGUUGGCAAUCUCUU-3'	dTdT
	siRNA_0005567_anty1	5'-AAGAGAUUGCCAACAAAAGGA-3'	dTdT
**Set 2**	siRNA_0005567_sens2	5'-GUUGGCAAUCUCUUCUCUGAA -3'	dTdT
	siRNA_0005567_anty2	5'-UUCAGAGAAGAGAUUGCCAAC-3'	dTdT

Table S3. Characterization of primers used for micro-RNAs evaluation.

Target name	Primer sense 5′→3′	Primer antisense 5′→3′	Mature micro-RNA sequence 5′→3′	miRBase ID
**hsa-miR-194-5p**	CAGCAGTGTAACAGCAACTCCA	TCAGTTTTTTTTTTTTTTTCCACAT	UGUAACAGCAACUCCAUGUGGA	MIMAT0000460
**hsa-miR-620**	ACAGTCGCAGATGGAGATAGATATAG	AGTCCAGTTTTTTTTTTTTTTTTATTTC	AUGGAGAUAGAUAUAGAAAU	MIMAT0003289
**hsa-miR-1270**	TGCTGGAGATATGGAAGAGCTG	GGTCCAGTTTTTTTTTTTTTTTACAC	CUGGAGAUAUGGAAGAGCUGUGU	MIMAT0005924
**hsa-miR-383-5p**	CACAGACAGCACTGCCTGGT	GTCACAGTTTTTTTTTTTTTTTCTGA	AGAUCAGAAGGUGAUUGUGGCU	MIMAT0000738
**hsa-miR-579-3p**	TCAGCAGTTCATTTGGTATAAACC	TCCAGTTTTTTTTTTTTTTTAATCG	UUCAUUUGGUAUAAACCGCGAUU	MIMAT0003244
**hsa-miR-548c**	TACCGCATCAAAAATCTCAATTAC	GTCCAGTTTTTTTTTTTTTTTGCA	CAAAAAUCUCAAUUACUUUUGC	MIMAT0003285
			**Reference sequence according to GeneBank**	
**RNU6-1 (U6)**	CTTCGGCAGCACATATACTAAAA	GAATTTGCGTGTCATCCTTG	NR_004394.1	-

Table S4. Characterization of primers used for transcripts evaluation – In-silico prediction.

Gene	Primer sense 5′→3′	Primer antisense 5′→3′
SETD5	GTGACAAGTGCAGGGGAATG	TAGGTGTGTGCTGTGTTGCT
KPNA1	ATGACCACCCCAGGAAAAGAG	TTTCTCCGCTTGAATAACTGCT
HBEGF	TGCTGTCATCTGTCTGTCTGC	GTCCCCAGCCGATTCCTTGAG
KDM5B	GGAGACAACGGAAGCCAGAA	CAGACATACAGGTCCACAGCA
TLN2	AGCAACTGAAGCCAAGAGCA	GATGGAGCCCACCTTTCCTT
IGF1R	GCCGACGAGTGGAGAAATCTG	TGGAGGTAGCCCTCGATCAC

Table S5. Characterization of primers used for transcripts evaluation – literature search.

Gene	Primer sense 5′→3′	Primer antisense 5′→3′	Reference publication – PubMed ID*
SOCS2	GGCCACCTGTCTTTGCCG	ACTTCCCCAGTACCATCCTGT	PMID: 36539807
IL6ST	GCATTGTGAACGAGGGGA	AACTTGTGTGTTGCCCATTC	PMID: 36620589
NR2F2	CTGTCCCATCGACCAGCAC	CTGCACCGCTTCCCGT	PMID: 33200800
MAPK1	CCGAGTGACGAGCCCATCG	AGAACTGGGAAGAAGAACACCG	PMID: 33987352
STAT1	TGTTTCATTTGCCACCATCCG	TCCCCGACTGAGCCTGATTA	PMID: 33194019
AKT2	GCTCCACAAGCGTGGTGAAT	GCAGGCAGCGTATGACAAAG	PMID: 31496625
SOX5	GAGTTTTCCCAACAAGCCTCA	TTGCCATCAACTTCCATTGTAT	PMID: 22396742
FOXA1	CCAGGATGTTAGGAACTGTGAA	GAGTAGGCCTCCTGCG	PMID: 35008751

***For detailed references and citation please refer to main manuscript.**
